# A Contemporary Approach to the Management of Asymptomatic Severe Aortic Stenosis

**DOI:** 10.3390/jcm15093405

**Published:** 2026-04-29

**Authors:** Parth V. Desai, Keerthi Gondi, Elizabeth Davis, Dantwan Smith, Alexandra V. Sykes, Michael E. Jessen, Lynn Huffman, Ahmed Zaghloul, Weiyi Tan, Ki Park, Dharam J. Kumbhani, Anthony A. Bavry, Amit Goyal

**Affiliations:** 1 Cardiovascular Medicine, PLLC, Davenport, IA 52803, USA; 2 CardioNerds, Baltimore, MD 21209, USA; 3 Division of Cardiology, Department of Internal Medicine, University of Texas Southwestern Medical Center, Dallas, TX 75235, USA; 4 Division of Cardiology, Department of Internal Medicine, Veterans Affairs North Texas Health Care System, Dallas, TX 75216, USA; 5 Division of Cardiology, Department of Internal Medicine, The University of Texas Medical Branch, Galveston, TX 77555, USA

**Keywords:** asymptomatic severe aortic stenosis, transcatheter aortic valve replacement, surgical aortic valve replacement, early intervention, watchful waiting, risk stratification, contemporary management

## Abstract

Asymptomatic severe aortic stenosis (AS) is characterized by a prolonged latent phase during which progressive valvular obstruction and myocardial remodeling may occur despite preserved left ventricular ejection fraction and the absence of overt clinical symptoms. Historically, management has favored watchful waiting until symptom onset or guideline-defined triggers emerge; however, recent randomized data challenge this conservative paradigm. This review summarizes the natural history, risk stratification, and contemporary management of asymptomatic severe AS, with a focus on emerging insights that inform the timing of intervention. We propose an individualized, contemporary framework for managing asymptomatic severe AS that integrates multimodal risk assessment, procedural risk, and shared decision-making, and we outline future directions aimed at refining patient selection and optimizing the personalized timing of intervention.

## 1. Natural History of Aortic Stenosis

Aortic stenosis (AS) is a progressive valvular disease characterized by obstruction of left ventricular (LV) outflow due to narrowing of the aortic valve orifice. It is one of the most commonly encountered heart valve lesions in developed countries, with a prevalence of 1–2% in adults ≥ 65 years old and approximately 12% in those >75 years old [1]. It can lead to compensatory changes in the LV that result in heart failure, poor cardiac output, and ultimately death when untreated [2]. The natural history of AS follows a prolonged latent phase during which the disease progresses gradually but remains asymptomatic, followed by a rapid decline once symptoms develop. The most common etiologies of AS are degenerative, rheumatic, and congenital, including bicuspid aortic valvopathy [2,3]. Progression of AS is associated with common cardiovascular risk factors such as hypertension, diabetes, and hyperlipidemia. It begins with fibrocalcific changes in the aortic valve leaflets, which result in thickening, reduced mobility, and progressive orifice obstruction, thereby causing LV pressure overload [2]. The underlying process resembles that of atherosclerosis, involving lipid accumulation, inflammation, and calcification. Over time, the LV compensates for the increased afterload through concentric hypertrophy, maintaining normal wall stress and cardiac output for years. Progression of AS is most commonly monitored using echocardiography, with specific criteria to delineate severity. Severe high-gradient AS is defined as aortic valve area ≤1.0 cm^2^, mean gradient ≥ 40 mmHg, and peak velocity ≥ 4 cm/s [4]. During the asymptomatic period, patients generally maintain preserved systolic function despite increasing transvalvular gradients. The rate of progression varies, but on average, the aortic valve area decreases by approximately 0.1 cm^2^ per year, and the mean gradient increases by approximately 7 mmHg per year [5]. This stage may last decades, and sudden death is exceedingly rare in the absence of symptoms or LV dysfunction. Regular echocardiographic surveillance is essential during this phase to monitor disease progression. Up to 50% of patients with asymptomatic AS progress to symptomatic AS over two years [5]. The emergence of symptoms is an ominous development in the natural history of AS, as most cases are fatal within 2–3 years of symptom emergence without valvular intervention [3]. The three classic symptoms include angina, syncope, and dyspnea, as expected of heart failure [2]. Each of these hallmark symptoms corresponds to distinct pathophysiologic consequences: angina from increased myocardial oxygen demand and subendocardial ischemia; syncope from fixed cardiac output during exertion; and dyspnea from elevated left-sided pressures and pulmonary congestion [2]. The onset of symptoms marks the transition from compensated hypertrophy to ventricular decompensation. Progression and symptom onset are influenced by several factors, including the severity of valve calcification, the rate of hemodynamic progression, age, and comorbidities such as renal dysfunction or metabolic syndrome [6]. Cardiovascular imaging, including computed tomographic valve calcium scoring and echocardiographic global longitudinal strain analysis, may enable earlier identification of patients at risk of rapid progression or subclinical LV dysfunction [7]. Understanding the natural history of AS underscores the importance of timely diagnosis and intervention. While medical therapy may alleviate symptoms or address comorbidities, no currently available medical treatment halts disease progression. Aortic valve replacement (AVR)—surgical or transcatheter—remains the only definitive therapy once symptoms or LV dysfunction develop.

## 2. Current Guideline Recommendations

The 2020 ACC/AHA Guideline for the management of patients with valvular heart disease provides recommendations on evaluation and appropriate timing of treatment for severe AS [8]. AVR is recommended in all symptomatic patients with severe AS, regardless of LVEF, including those with severe high-gradient AS (Stage D1; Class I), low-flow low-gradient AS with reduced LVEF (Stage D2; Class I), and low-flow low-gradient AS with normal LVEF (Stage D3; Class I). Recommendations are more nuanced for the management of asymptomatic severe AS (Stage C). AVR is strongly indicated for asymptomatic patients with LVEF < 50% (Class I) and for those undergoing cardiac surgery for other indications (Class I). There is additionally a recommendation for AVR in patients with asymptomatic severe AS with an abnormal exercise test (Class IIa), elevated serum B-type natriuretic peptide (BNP) level (Class IIa), very severe AS with transaortic velocity of ≥5 m/s (Class IIa), and rapid progression with an increase in aortic velocity ≥ 0.3 m/s per year (Class IIa). The 2025 ESC/EACTS Guidelines are similar in providing a recommendation for AVR in all patients with symptomatic severe AS (Class I), in asymptomatic patients with LVEF < 50% (Class I), and in asymptomatic patients with high-risk features such as very high-gradient, rapid progression, elevated biomarkers, abnormal exercise testing, and severe valve calcification (Class IIa) [9]. For patients who are truly asymptomatic with normal LVEF and no additional high-risk parameters, there is no current guideline recommendation on the role of early AVR prior to the development of symptoms. Multiple clinical features and parameters are useful for risk-stratifying patients with asymptomatic severe AS to inform clinical decision-making regarding AVR timing and the frequency of clinical surveillance.

## 3. Risk Stratification Tools for Asymptomatic Severe Aortic Stenosis Patients

**Exercise Stress Test and Exercise Stress Echocardiography:** Exercise stress testing remains a cornerstone for risk stratification in patients with asymptomatic severe AS. It is contraindicated in symptomatic severe AS due to the risk of hemodynamic compromise, including syncope, ventricular tachycardia, and death [8,10]. In apparently asymptomatic individuals, gradual activity limitation may mask symptoms that become apparent during exertion; thus, exercise testing may unmask latent symptoms and guide timing of AVR [1,8]. The 2020 ACC/AHA guidelines provide a Class IIa recommendation for exercise testing in asymptomatic patients with severe AS to assess physiological changes with exercise and confirm the absence of symptoms [8]. Abnormal responses—including reduced exercise tolerance (normalized for age and sex) or a ≥10 mmHg fall in systolic blood pressure from baseline to peak exercise—identify patients at increased risk for symptom development and adverse outcomes, warranting consideration for AVR [1].

Beyond symptom elicitation, exercise stress echocardiography (ESE) provides incremental prognostic value by enabling dynamic assessment of valve hemodynamics [11,12]. An exercise-induced increase in mean gradient > 20 mmHg independently predicts clinical events (HR 3.83; 95% CI 2.16–6.67) [12]. The development of exercise-induced pulmonary hypertension (systolic pulmonary arterial pressure > 60 mmHg) at peak exercise also provides incremental prognostic value [11]. A 2025 prospective study showed that serial annual ESE safely delayed AVR by a mean of 2.93 years while identifying high-risk patients, with no cardiac-related deaths during follow-up [13]. Accordingly, the incorporation of ESE into the evaluation of asymptomatic severe AS enhances individualized risk stratification and may refine the timing of AVR, particularly in physically active patients where resting parameters may underestimate disease severity.

**Transthoracic Echocardiogram:** Multiple echocardiographic parameters are useful for risk stratification in patients with asymptomatic severe AS. A primary distinction is the presence of LV systolic dysfunction or very severe or critical AS, defined as those with a very high transvalvular gradient (aortic velocity ≥ 5 m/s or mean pressure gradient ≥ 60 mmHg). LV dysfunction can occur in severe AS due to progressively worsening afterload and implies a high risk of impending symptom onset. In an international, multicenter, prospective registry of 1763 patients with AS, the strongest independent predictors of cardiovascular mortality were LVEF < 60% (HR 4.47) and peak aortic velocity ≥ 5 m/s (HR 6.31) [14]. The presence of LV systolic dysfunction, serial decline in LVEF, or very severe AS suggests a markedly elevated impending risk of mortality, and consideration of earlier AVR is recommended even if symptoms are not present [8]. There has been growing interest in the role of LV global longitudinal strain (LV GLS) in risk stratification for severe AS. GLS by speckle-tracking echocardiography reflects longitudinal myocardial shortening, and an abnormal LV GLS (greater than −16%) indicates early contractile impairment and subclinical myocardial dysfunction, often preceding symptom onset. Multiple observational analyses have suggested that LV GLS and other parameters of LV myocardial health are strongly predictive of symptom development and poor outcomes [15,16,17]. In an individual participant data meta-analysis of 1067 patients with asymptomatic severe AS and normal LVEF, impaired LV GLS was associated with 2.5-fold reduced survival [18]. LV GLS is not currently reflected in guideline recommendations, but overall measures of LV health and subclinical LV dysfunction may aid clinical decision-making for asymptomatic severe AS, as patients with impaired LV GLS may be considered higher risk and therefore suitable for frequent echocardiographic monitoring or earlier AVR.

**Serum biomarkers:** Natriuretic peptides, including BNP and the prohormone N-terminal pro B-type natriuretic peptide (NT-proBNP), are released from the myocardium in response to ventricular or atrial stretch [19]. Studies evaluating BNP and NT-proBNP in AS have demonstrated associations with echocardiographic findings of severe AS (peak velocity, mean gradient, aortic valve area), symptoms, risk of symptom onset, risk of death, a need for AVR, cardiovascular hospitalization, and abnormal exercise hemodynamics [19,20,21,22,23]. Elevated levels can indicate myocardial dysfunction and subclinical heart failure, and natriuretic peptide elevation generally occurs before symptoms or overt heart failure develop. Multiple studies have verified that BNP is a powerful predictor of symptom-free survival [20,24,25]. In a large multicenter registry, incremental increases in BNP level were strongly associated with AS-related adverse events in patients with asymptomatic severe AS, and patients with BNP levels within the normal range had a low event rate of 2.1% at 1 year [26]. This finding was also noted in a post hoc sub-study of the SEAS trial, in which patients with asymptomatic severe AS and normal NT-proBNP levels had low rates of adverse events and mortality [27]. For this reason, current guidelines give a Class IIa recommendation to consider AVR for asymptomatic severe AS patients with elevated BNP (>3× upper limit of normal), accounting for age- and sex-related variation in reference values [8]. Natriuretic peptides can thus be a valuable adjunct tool in asymptomatic severe AS to assess the risk of symptom onset and underlying heart failure, and serial measurements may provide additional prognostic value by identifying rising trends associated with disease progression [28].

Troponin elevation reflects the release of cardiac-specific contractile filaments in the setting of myocardial necrosis or cell membrane injury. Hemodynamic obstruction from AS can lead to increased myocardial oxygen demand, leading to oxidative stress, cardiac injury, and troponin release [29]. In an observational study of patients with AS, high-sensitivity troponin I levels were associated with the degree of myocardial stress, as indicated by LV hypertrophy and late gadolinium enhancement myocardial fibrosis on cardiac magnetic resonance imaging. This study also showed that markedly elevated high-sensitivity troponin I (≥10.7 ng/L) was associated with a nearly twofold risk of AVR and cardiovascular death, and this association was independent of patient age, sex, LV systolic function, or aortic valve severity [30]. When used in conjunction with NT-proBNP, high-sensitivity troponin has been shown to improve the prediction of mortality in asymptomatic patients treated conservatively or who underwent SAVR or TAVR [31,32].

**Cardiac MRI:** Cardiac MRI (CMR) is increasingly used for risk stratification of asymptomatic severe AS. CMR can accurately describe and quantify the extent of LV remodeling and myocardial fibrosis associated with AS. Multiple prospective studies have demonstrated the powerful prognostic value of CMR-derived assessment of myocardial fibrosis in predicting outcomes in asymptomatic AS [33,34,35]. In a prospective multicenter cohort of 457 patients, increased diffuse interstitial fibrosis was independently associated with mortality and provided incremental prognostic value when added to traditional parameters of AS severity [34]. Additionally, CMR can effectively identify concomitant cardiac amyloidosis, which is present in ~5–10% of patients with severe AS (particularly low-flow, low-gradient AS) and is associated with a worse AS-related prognosis and is itself treatable [36,37,38]. CMR can therefore be informative in risk-stratifying asymptomatic severe AS by assessing myocardial fibrosis; diffuse fibrosis may represent a high-risk feature that would prompt earlier AVR or more frequent surveillance in asymptomatic patients. However, CMR is not currently routinely recommended for patients with severe AS, and further studies are needed to assess the clinical outcomes of CMR-guided AVR decision-making in asymptomatic patients.

## 4. Surgical Aortic Valve Replacement in Asymptomatic Severe Aortic Stenosis

Before the transcatheter era, the risks of open-heart surgery justified a conservative “watchful waiting” approach to asymptomatic severe AS until symptoms or LV dysfunction developed. However, this silent phase is not benign; patients face increased mortality, cardiac comorbidities, and subclinical decline in functional capacity [5,39,40]. Emerging risk stratification tools, including biomarkers, LV strain, and exercise echocardiography, have helped identify patients who may benefit from earlier intervention [41,42,43]. These observations prompted the randomized evaluations of early surgical intervention with two key trials—RECOVERY and AVATAR.

The RECOVERY trial randomized 145 patients with very severe asymptomatic AS (aortic valve area ≤ 0.75 cm^2^, peak velocity ≥ 4.5 m/s, or mean gradient ≥ 50 mmHg) and preserved LV ejection fraction (LVEF ≥ 50%) to early surgical aortic valve replacement (SAVR) or conservative management [44,45]. Over a median follow-up of 12 years, early SAVR significantly reduced the primary composite endpoint of operative mortality or cardiovascular death (3% vs. 24%; HR 0.10, *p* = 0.002), with cumulative incidence of 1% vs. 19% at 10 years [45]. All-cause mortality was also lower in the early surgery group (15% vs. 32%; HR 0.42), and the Kaplan–Meier curves showed sustained separation without convergence, underscoring the durability of benefit. Importantly, the incidence of repeat aortic valve surgery was similar between groups, suggesting that early intervention did not increase long-term prosthetic valve complications [45]. Although the trial demonstrated that early surgery improves outcomes in selected patients, its generalizability remains limited by a relatively young cohort (mean age ≈ 64 years), a high prevalence of bicuspid valves (61%), lack of routine exercise testing to confirm asymptomatic status, and a single-country design with excellent surgical outcomes.

The AVATAR trial was a multicenter, randomized controlled trial that enrolled 157 low-risk patients (mean age 67 years; LVEF ≥ 50%) with severe asymptomatic AS, as confirmed by a negative exercise stress test [46]. Participants were randomized to early SAVR or conservative management, with AVR deferred until symptom onset or guideline-defined triggers occurred. The primary endpoint was a composite of all-cause death, acute myocardial infarction, stroke, or unplanned heart failure hospitalization. During a median follow-up of 32 months, early AVR significantly reduced the primary composite outcome compared with conservative management (HR 0.46; 95% CI 0.23–0.90; *p* = 0.02), with an operative mortality of 1.4%. At extended follow-up (median 63 months), this benefit persisted, with 23.1% of patients in the early-surgery group versus 46.8% in the conservative group reaching the primary endpoint (HR 0.42; 95% CI 0.24–0.73; *p* = 0.002) [47]. All-cause mortality (HR 0.44; *p* = 0.012) and heart failure hospitalizations (HR 0.21; *p* = 0.007) were also significantly lower with early intervention.

These results indicate that early SAVR in well-defined asymptomatic severe AS with preserved LV function significantly reduces major adverse cardiovascular events and mortality compared with watchful waiting. These findings mark a transition from a reactive, symptom-driven strategy to a more proactive, preventive approach in selected low-risk patients. Collectively, RECOVERY and AVATAR laid the foundation for early intervention, but their focus on low-risk surgical candidates left open questions about their applicability to older or higher-risk populations. These uncertainties led to transcatheter trials such as EARLY TAVR and EVOLVED, which aimed to expand and refine this preventive paradigm in the contemporary era.

## 5. Transcatheter Aortic Valve Replacement in Asymptomatic Severe Aortic Stenosis

Following the results of RECOVERY and AVATAR, subsequent transcatheter trials aimed to evaluate whether the same preventive strategy could be extended to an older, low- to intermediate-surgical-risk population more representative of contemporary TAVR candidates. The two contemporary randomized controlled trials addressing this question are EARLY TAVR and EVOLVED.

The EARLY TAVR trial was a multicenter, randomized study that enrolled 901 patients (mean age 76 years; 35% women) with asymptomatic severe AS and a preserved LVEF (≥50%) [48]. All patients were classified as low surgical risk (STS < 3%). Importantly, asymptomatic status was confirmed by detailed clinical evaluation, including mandatory exercise testing when clinically appropriate, to exclude those with exertional limitation or unrecognized symptoms. Patients were randomized in a 1:1 ratio to early TAVR using a balloon-expandable valve (SAPIEN 3 or SAPIEN 3 Ultra, Edwards Lifesciences) or to clinical surveillance, with crossover permitted upon symptom onset or other guideline triggers. The primary composite endpoint—all-cause death, stroke, or unplanned cardiovascular hospitalization—occurred in 26.8% of the early-TAVR group versus 45.3% of the surveillance group over a median follow-up of 3.8 years (HR 0.50; 95% CI 0.40–0.63; *p* < 0.001). The benefit was mainly driven by fewer cardiovascular hospitalizations, with no increase in stroke or mortality. Procedural safety was consistent with prior low-risk TAVR trials (new pacemaker 12.6%, disabling stroke 2.9%). Early intervention also led to earlier and sustained improvements in quality-of-life scores.

In a sub-study from EARLY TAVR, Lindman et al. showed that, in patients with asymptomatic severe AS, the benefit of early TAVR over clinical surveillance was consistent regardless of baseline LV function, as defined by global longitudinal strain, LV mass, and left atrial volume, as well as cardiac biomarkers [49]. These findings suggest that baseline measurable attributes of LV health have limited value in guiding the timing of intervention, although delayed treatment was associated with progressive LV remodeling and worsening LV health during follow-up [50].

The EVOLVED (Early Valve Replacement Guided by Biomarkers of LV Decompensation in Asymptomatic Patients with Severe Aortic Stenosis) trial was a multicenter, open-label, randomized controlled trial designed to assess whether early AVR guided by evidence of LV decompensation improves outcomes in asymptomatic severe AS [51]. A total of 224 patients (mean age, 73 years; 28% women) with severe AS (aortic valve area ≤ 1.0 cm^2^ or indexed ≤ 0.6 cm^2^/m^2^, mean gradient ≥ 40 mmHg, or peak velocity ≥ 4.0 m/s) and preserved LVEF (≥50%) were enrolled from 24 centers across the United Kingdom and Australia. All participants were clinically asymptomatic according to physician assessment and standardized questionnaires; however, exercise testing was not mandatory to confirm asymptomatic status, which distinguishes this study from other early-intervention trials. A unique feature of EVOLVED was its cardiac imaging-driven selection strategy. Eligibility required evidence of mid-wall late gadolinium enhancement (LGE) on CMR, reflecting myocardial fibrosis and subclinical LV decompensation. This design aimed to identify patients at higher biological risk despite the absence of symptoms, under the hypothesis that myocardial fibrosis represents a point of irreversible myocardial remodeling that could benefit from earlier valve replacement. Patients were randomized to early AVR (either TAVR or SAVR, determined by the local Heart Team) or standard guideline-directed surveillance, with intervention deferred until the development of symptoms, LV systolic dysfunction, or other conventional indications. Approximately three-quarters (75%) of patients in the early-intervention arm underwent surgical AVR, while the remainder received TAVR, reflecting procedural selection based on age and anatomic suitability. The primary endpoint of EVOLVED was a composite of all-cause death or unplanned AS-related hospitalization. Over a median follow-up of 24 months, this endpoint occurred in 18% of the early-intervention group versus 23% of the surveillance group (HR 0.79, 95% CI 0.44–1.43, *p* = 0.44), indicating no significant difference between strategies. There were also no significant differences in individual components, including all-cause mortality (7% vs. 9%), cardiovascular death, or heart failure hospitalization. Despite the neutral primary outcome, early intervention was associated with a lower rate of unplanned AS-related hospitalization (6% vs. 17%) and fewer NYHA class II–IV symptoms at 12 months (20% vs. 38%), suggesting improved functional status and symptom burden. Secondary analyses showed favorable trends in LV mass regression and reduction in natriuretic peptide levels, although these did not translate into measurable differences in major clinical outcomes during the relatively short follow-up. Procedural mortality was 0%, and serious adverse events were rare, confirming the safety of early valve replacement. Event rates were lower than anticipated, limiting statistical power, and the predominance of surgical over transcatheter procedures meant that the trial did not fully assess the role of early TAVR in this population.

## 6. Why Did EARLY TAVR and EVOLVED Show Different Topline Results?

At first glance, the EARLY TAVR and EVOLVED trials appear to provide conflicting conclusions regarding the putative benefit of early intervention in asymptomatic severe AS. However, these differing outcomes are best explained by variations in trial design, patient selection, procedural strategy, and endpoint structure rather than a fundamental disagreement on the value of early treatment (Table 1).

*Trial Size and Power*: The EARLY TAVR trial was a large, adequately powered study enrolling over 900 patients, whereas EVOLVED was smaller and statistically underpowered (*n* = 224 enrolled out of the initially planned sample size of 356 patients), partly due to COVID-19–related enrollment challenges. The smaller sample size and lower event rate in EVOLVED limited its ability to detect a treatment effect, even if one was present [48,51].

*Patient Selection and Myocardial Biology*: The divergent outcomes of EARLY TAVR and EVOLVED may also be explained by differences in myocardial disease stage at the time of intervention, driven by deliberate differences in patient selection. Progressive AS is characterized by a continuum of myocardial remodeling, evolving from adaptive hypertrophy to replacement fibrosis and irreversible myocardial injury. While valve replacement effectively relieves afterload and prevents further damage, its capacity to reverse established fibrosis is limited.

EARLY TAVR enrolled a broad population of older patients with asymptomatic severe AS and preserved LVEF, representative of contemporary transcatheter valve candidates and not selected for advanced myocardial injury. In this early disease phase, intervention occurred before irreversible myocardial remodeling predominated, potentially preventing clinical decompensation and preserving myocardial function.

In contrast, EVOLVED selectively enrolled patients with evidence of established subclinical myocardial damage, including mid-wall myocardial fibrosis on cardiac magnetic resonance imaging. In this biologically advanced cohort, early valve intervention reduced valve-related hospitalizations and improved functional status but did not translate into reductions in all-cause or cardiovascular mortality, suggesting that intervention may have occurred beyond a critical “window of reversibility,” perhaps as indicated by the presence of maladaptive myocardial fibrosis used to gate entry into this trial.

*Timing of Intervention*: In EARLY TAVR, patients underwent transcatheter valve replacement within a median of two weeks following randomization, minimizing the delay between diagnosis and treatment. In EVOLVED, the median time from randomization to intervention was approximately five months, long enough for disease progression and attenuation of the early-protection effect.

*Generalizability and Sex Distribution*: The EVOLVED cohort had a lower proportion of female participants, potentially limiting generalizability and underrepresenting sex-specific patterns of LV remodeling and fibrosis progression that may influence outcomes.

In summary, the apparent discrepancy between EARLY TAVR and EVOLVED arises from key differences in sample size, patient phenotype (general asymptomatic vs. fibrosis-positive), and the timing of intervention. EARLY TAVR supports early transcatheter intervention in a broader asymptomatic severe AS population with low procedural risk, whereas EVOLVED suggests that once myocardial fibrosis is established, the opportunity for maximal benefit may be narrower and more difficult to demonstrate statistically. Collectively, these studies reinforce a convergent message: early preventive intervention in well-selected asymptomatic patients can reduce hospitalizations, improve functional status, and maintain quality of life—even if a survival advantage has not yet been consistently demonstrated over a near-term follow-up period.

## 7. Ongoing Trials

Beyond these pivotal studies, the EASY-AS trial (Early Valve Replacement in Severe Asymptomatic Aortic Stenosis; NCT04204915) [52,53] is an ongoing randomized study evaluating early AVR (TAVR or SAVR) versus conservative management in a broader asymptomatic population. This trial includes exercise-testing protocols similar to those used in EARLY TAVR and aims to clarify whether early intervention provides consistent benefit across age and procedural subgroups.

## 8. Implications for Clinical Practice

Recent randomized trials have increased interest in earlier intervention for patients with asymptomatic severe AS. However, current clinical practice remains guided by established recommendations, which reserve AVR for patients with symptoms, LV systolic dysfunction (LVEF < 50%), abnormal exercise testing, or other high-risk features as detailed above [1,8]. Regulatory indications similarly limit TAVR to symptomatic severe AS, and early intervention in asymptomatic patients therefore remains selective.

The absence of a demonstrated survival benefit should not be interpreted as the absence of a clinically meaningful benefit. Patient-centered outcomes, such as heart failure hospitalizations, functional status, and quality of life, are important considerations for patients and health systems. In this context, EARLY TAVR demonstrated reductions in unplanned cardiovascular hospitalizations and improvements in quality-of-life measures, while EVOLVED showed that early valve replacement can be performed safely in selected imaging-positive cohorts. Together, these findings suggest that selected patients may derive benefit from earlier intervention even in the absence of a clear mortality advantage.

### 8.1. Limitations of Current Evidence and Bioprosthetic Valve Durability Concerns

A key limitation of the current evidence is the relatively short follow-up duration, typically 12–24 months. This period is substantially shorter than the timeframe during which structural valve degeneration (SVD) is expected to occur in bioprosthetic valves, whether surgically implanted or via TAVR. Early valve replacement, therefore, exposes the patient to the finite durability of a bioprosthetic valve at an earlier time point. Because SVD may require repeat AVR, it remains uncertain whether early clinical benefits will be sustained over the lifetime of the patient who was treated early for asymptomatic severe AS. Key tenets of lifelong management of AS weigh even more heavily when considering early AVR in this low risk and currently asymptomatic population, expectedly with longer longevity [54,55].

Emerging long-term data from low-risk TAVR trials provide important insights. Extended follow-up from the Evolut Low Risk trial demonstrated higher rates of reintervention in the TAVR arm at 6–7 years (9.8% vs. 6.0% at 7 years; *p* = 0.02), largely driven by progressive aortic regurgitation (5.6% vs. 1.6%; *p* < 0.001), highlighting a potential late durability signal with this particular self-expanding platform [56]. In contrast, the PARTNER 3 trial at 7 years showed similar rates of bioprosthetic valve failure (6.9% vs. 7.3%) and aortic valve reintervention between balloon-expandable TAVR and surgery [57], suggesting that durability profiles may be device-specific rather than a class effect.

### 8.2. Lifetime Management Considerations

These observations have important implications for younger and asymptomatic patients considered for early TAVR. Early implantation effectively initiates the “bioprosthetic clock” sooner, increasing the likelihood that patients will require multiple valve interventions over their lifetime. This necessitates a structured lifetime management framework, incorporating anticipation of future valve-in-valve procedures, preservation of coronary access, and mitigation of risks such as patient–prosthesis mismatch and coronary obstruction [54,55,58]. Anatomic factors—including annular size, sinus of Valsalva dimensions, and coronary height—are central to this planning and should be considered at the time of initial intervention.

### 8.3. Patient Selection for Early AVR Intervention

**Figure 1:**
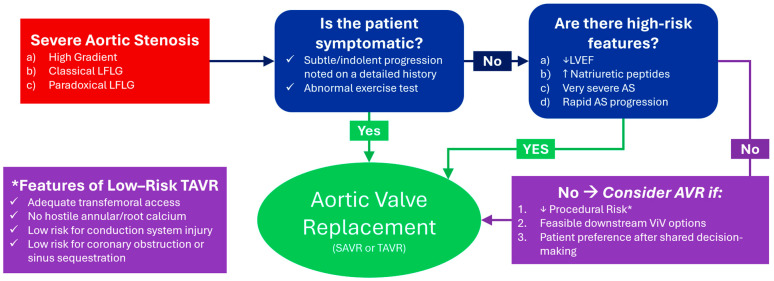
Management of Asymptomatic Severe Aortic Stenosis. This flowchart proposes a framework for the contemporary management of asymptomatic severe aortic stenosis based on the authors’ expert opinion. Acronyms: AS, aortic stenosis; AVR, aortic valve replacement; LFLG, low-flow low-gradient; LVEF, left ventricular ejection fraction; SAVR, surgical aortic valve replacement; TAVR, transcatheter aortic valve replacement.

In clinical practice, evaluation of patients described as having “asymptomatic severe AS” should begin with confirmation that guideline-based indications for intervention are not already present (Figure 1). This requires careful assessment for subtle or gradual symptom onset and progression, often incorporating collateral history from family members, as well as objective evaluation with exercise testing when appropriate and attention to LVEF, AS severity, and biomarkers as recommended by current guidelines.

For patients who are truly asymptomatic and do not meet guideline-recommended high-risk criteria, consideration of early valve replacement—transcatheter or surgical—should be limited to carefully selected individuals and guided by shared decision-making. Such patients should have low procedural risk. For TAVR candidates, this includes favorable transfemoral access, absence of high-risk annular, aortic root, or LV outflow tract calcium, a low risk of coronary obstruction or sinus sequestration, and a low anticipated risk of conduction system injury [54]. Patients with baseline right bundle branch block, extensive nodular annular calcium, or the need for alternative access strategies are less suitable for a preventive transcatheter approach. In addition, anatomic features should support future reintervention, including an adequate index valve size and aortic root dimensions that preserve coronary access and reduce the risk of future valve-in-valve procedures [54,58].

Overall, the available data support a selective, individualized approach to early intervention in asymptomatic severe AS rather than routine preventive valve replacement. Additional randomized data with longer follow-up and greater clarity around patient selection will be essential to guide shared decision-making, particularly regarding long-term valve durability, reintervention risk, and alignment with individual patient goals (Figure 1).

## 9. Future Perspectives

Risk stratification in asymptomatic severe AS remains limited when based primarily on symptoms and LVEF, which often fail to detect early myocardial decompensation. Growing evidence highlights the heterogeneous natural history of asymptomatic AS and the need for improved tools to identify patients at higher risk of disease progression. Current strategies—including biomarkers, advanced echocardiographic parameters, and cardiac imaging—aim to define this subgroup but, when used individually, have limited predictive performance. GLS provides incremental prognostic value by identifying early systolic dysfunction despite preserved LVEF. Ejection dynamic parameters such as acceleration time and the acceleration time–to–ejection time (AT/ET) ratio offer complementary insight into valve obstruction physiology, particularly in low-flow or discordant states. Biomarkers, including natriuretic peptides and high-sensitivity troponin, reflect myocardial stress and injury, while cardiac magnetic resonance imaging can detect myocardial fibrosis as a marker of advanced subclinical disease. However, no single parameter reliably identifies asymptomatic patients who will progress rapidly or clearly benefit from early intervention.

A key unmet need is the development of a refined, validated predictive risk score that can reliably identify patients with asymptomatic severe AS at the highest risk of progression and adverse outcomes. Future research should focus on prospective validation of multimodal markers, the establishment of clinically actionable thresholds, and the integration of echocardiography, biomarkers, cardiac MRI, and functional testing into composite risk models to better inform the individualized timing of AVR as procedural risk continues to decline.

## Figures and Tables

**Table 1:** Key Differences in EARLY TAVR and EVOLVED Trials. This table highlights key differences between the EARLY TAVR and EVOLVED trials. Acronyms: AS, aortic stenosis; CMR, cardiac magnetic resonance imaging; CV, cardiovascular; HF, heart failure; NYHA, New York Heart Association; SAVR, surgical aortic valve replacement; TAVR, transcatheter aortic valve replacement.

	EARLY TAVR	EVOLVED
Type of Valve Replacement	100% TAVR	~75% SAVR
**Trial Size & Power**	Large trial (>900 patients) Adequately powered to detect treatment differences	Small and underpowered trial (*n* = 226) Lower event rates limited ability to detect benefit
**Patient Selection**	Broad asymptomatic severe AS population Older, typical real-world TAVR candidates	Mid-wall myocardial fibrosis on CMR Represents subclinical myocardial injury May have passed the “window of reversibility”
**Timing of Intervention**	TAVR performed ~2 weeks after randomization Minimizes progression between randomization and treatment	AVR performed ~5 months after randomization Long delay allowed disease progression May blunt early-intervention benefits
**Primary Endpoint**	Composite: death, stroke, or unplanned CV hospitalization	Composite: all-cause death or AS-related hospitalization
**Secondary Outcomes**	Reduced HF hospitalization Improved quality of life	Lower unplanned AS-related hospitalization Fewer NYHA II–IV symptoms at 12 months
**Generalizability**	Representative sex distribution	Fewer female participants → limits generalizability and may miss sex-specific fibrosis patterns

## Data Availability

No new data were created or analyzed in this study.

## Abbreviations

The following abbreviations are used in this manuscript:

AS	Aortic Stenosis
AVR	Aortic Valve Replacement
BNP	B-type natriuretic peptide
ESE	Exercise Stress Echocardiography
LV	Left Ventricle
LVEF	Left ventricular ejection fraction
NTproBNP	N-terminal pro B-type natriuretic peptide
SAVR	Surgical aortic valve replacement
SVD	Structural valve degeneration
TAVR	Transcatheter aortic valve replacement
